# Decarbonization: examining the role of environmental innovation versus renewable energy use

**DOI:** 10.1007/s11356-022-18686-1

**Published:** 2022-02-23

**Authors:** Bhagaban Sahoo, Deepak Kumar Behera, Dil Rahut

**Affiliations:** 1 Department of Economics, Anandapur College, Department of Education, Government of Odisha, Anandapur, Odisha 758021 India; 2 Department of Commerce, Manipal Academy of Higher Education, Manipal, Karnataka 576104 India; 3 Asian Development Bank Institute, Tokyo, 100-6008 Japan

**Keywords:** Decarbonization, CO_2_ emissions, Environmental innovation, Environmental technology, Globalization, Renewable energy, Developing Asia economies, O33, O44, O53, Q01, Q53, Q54, Q55

## Abstract

Climate change resulting from a rapid increase in greenhouse gas (GHG) emissions is adversely affecting humanity. If the GHG emission continues to rise at the current pace, humanity will face severe consequences and reverse all the progress made. This paper, therefore, uses relevant data from 14 developing countries in Asia from 1990 to 2018 to examine the potential impact of environmental innovation on CO_2_ emissions by controlling globalization, urbanization, and economic growth. The number of environmental-related technology patents is used as a measure of environmental innovation. We employed a panel long-run regression model — FMOLS, PCSE, and FGLS to estimate the elasticity of CO_2_ emissions. For causal association among variables, we used Dumitrescu-Hurlin Granger causality tests. Our results show that renewable energy consumption and globalization have a significant impact in reducing CO_2_ emissions, while environmental technology innovations play a meager role in reducing emissions and only when economic growth support those type of investment. Furthermore, we found urbanization, oil consumption, and economic growth is detrimental to the environment, which is also evident in past studies. Therefore, countries should invest in renewable energy and environmental innovation aligned with the growth to reduce GHG emissions.

## Introduction

Economic growth accompanied by rapid urbanization, industrialization, digitalization, and technology improvement and innovation has dramatically improved the well-being of the people in the last century (Allena and Fracchia 2017; Ahmed et al. 2019; Brandão Santana et al. 2015). However, economic growth has also led to the dramatic increase in CO_2_ emissions, destruction of the environment, and biodiversity loss; some of the damages may take several decades to reverse, while others may not be reversible. Several existing literatures provide evidence on the long-run and short-run relationship between economic growth, energy consumption, and environmental degradation (Attiaoui et al. 2017; Begum et al. 2015; Chen et al. 2019; Jafari et al. 2012; Ito 2017).

Since the declaration of United Nations (UN) Sustainable Development Goals (SDGs), reduction of environmental pollution through the adoption of green technology (i.e., renewable energy consumption and innovation of new energy sources) is one of the goals for developing countries (Brandão Santana et al. 2015; Zhang et al. 2017; Allena and Fracchia 2017; Ding et al. 2021. Recent studies show that innovation in environmental technology and increased use of/switching to green energy has played a crucial role in reducing GHG emissions and environmental pollution (Álvarez-Herránz et al. 2017; Chen and Lee 2020; Dauda et al. 2019; Khattak et al. 2020; Mensah et al. 2018; Töbelmann and Wendler 2020). Reducing environmental pollution has a positive impact on health and reduces the health care expenses of households (Balakrishnan et al. 2019; Rahut et al. 2017; Apergis et al. 2018). Therefore, investing in technology to reduce environmental pollution and GHG emissions would have a multifaceted impact on human well-being. Furthermore, the influence of green energy technology on reducing environmental pollution is indirectly influenced by economic globalization (Acheampong et al. 2019; Sabir and Gorus 2019; Salahuddin et al. 2019; Shahbaz et al. 2018).

Developing Asia is experiencing rapid economic growth, industrialization, and economic transformation and is considered a significant emitter of GHG emissions (Hanif et al. 2019; Hashmi et al. 2021; Lu et al. 2017; Mohsin et al. 2021). Rapid growth and economic transformation through increased consumption of non-renewable energy, less efficient technology, exploitation of natural resources, and limited investment in environmental protection and restoration (Ahmed et al. 2022; Lee et al. 2022) has significantly worsened the environmental degradation process. Past studies argue that higher economic growth and globalization could lead to technological innovation in the energy sector, resulting in reducing environmental pollution (Zaidi et al. 2019a, b; Chaudhry et al. 2021; Abid et al. 2021).

**Fig. 1:**
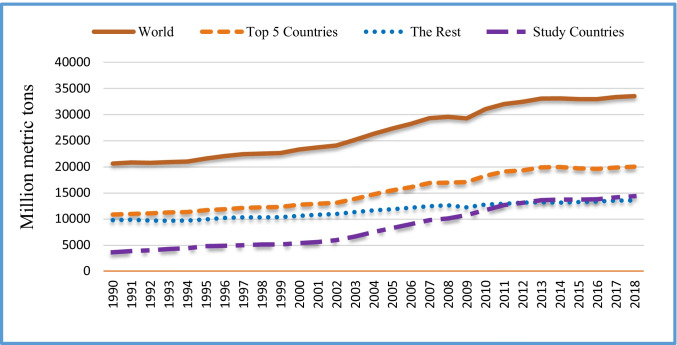
Global carbon-dioxide (CO_2_) emission trends

Figure 1 shows the global CO_2_ emission trends from 1990 to 2018 for the top five emitter countries,^1^ countries used in this study,^2^ the rest (excludes top 5 countries and countries not included in this study), and global. Figure 1 exhibits that CO_2_ emission is increasing rapidly; while the emission of the top five countries is plateauing, the emissions from 14 countries used in this study is rising and might surpass other countries in the coming years. The energy sector contributes to about 73.2% of the GHG emission while agriculture, forestry, and land use contribute 18.4%, the direct industrial process contributes 5.2%, and waste contributes 3.2% (Olivier and Peters 2020).

Against this backdrop, the current study examines two specific research questions: first, whether green energy (i.e., renewable energy) and green technology innovation (i.e., number of the environmental-related patent) reduce CO_2_ emissions per capita and its elasticity. Secondly, how much economic transformation-related factors such as urbanization, economic growth, oil consumption, and economic globalization affect the GHG emission using the data from 14 developing countries covering 1990 to 2018. To the best of our knowledge, this is one of the comprehensive studies in Asia that investigate the effects of green technology innovation on reducing GHG emissions. Few studies have used renewable energy and green innovation as a contributing factor for reducing environmental pollution but have not precisely adopted green technology patients to examine the relationships (Abid et al. 2021; Ahmed et al. 2022).

Furthermore, our study also use advanced econometric models to explore both elasticity and causality among the variables. We employed a panel long-run regression model — FMOLS, PCSE, and FGLS to estimate the elasticity of CO_2_ emission. For causal association among variables, we used Dumitrescu-Hurlin Granger causality tests.

The current paper is divided into five sections, including the “Introduction”. “Review of literature” discusses the past literature on possible factors responsible for the environmental deterioration and its likely impact on society and the economy. “Methodology” discusses the data and empirical methods. “Results and discussion” provides results and discussions. Finally, “Conclusions and policy implications” contains the conclusion and policy suggestions, and limitations.

## Review of literature

### Environmental technologies and CO_2_ emissions

Table 1 presents the literature that examines the nexus between environmental technologies and CO_2_ emission. Studies from China argue that carbon-free energy technologies patents, innovation in the existing technologies, and progress in domestic research and development have reduced carbon intensity and carbon emissions (Wang et al. 2012; Guo et al. 2017; Liang et al. 2019; and Luan et al. 2019; Shahbaz et al. 2020). Studies from developed countries (i.e., OECD, EU) argue that environmental patents and technological innovation significantly reduce carbon emission (Mensah et al. 2018; Cho and Sohn et al. 2018; and Töbelmann and Wendler 2020). Some studies from BRICS found that environmental innovation improves environmental quality in the long run and is moderated mainly by consumption of renewable energy (Khattak et al. 2020; and Erdogan 2021), while other studies find that environmental patent increases CO_2_ emissions due to barriers of patent diffusion (Cheng et al. 2019). Few countries from Asia–Pacific also support that technological innovation reduces environmental pollution, especially in the long run (Salman et al. 2019; Chaudhry et al. 2021).

**Table 1:** Environmental technologies and CO_2_ emissions

Authors	Period	Region/country	Methods	Findings
Wang et al. (2012)	1997–2008	30 Chinese provinces	GMM	Patents for carbon-free energy technologies play an important role in reducing CO_2_ emissions
Su and Moaniba (2017)	1976–2014	70 countries	Panel techniques	Climate change-related innovations reduce CO_2_ emissions from solid fuel and other greenhouse gas emissions
Guo et al. (2017)	2011–2012	China provinces	SEM	Technological innovation reduces carbon intensity
Mensah et al. (2018)	1990–2014	28 OCED	STIRPAT	Technological innovation and R&D contribute to lower environmental emission where GDP per capita induces Co_2_ emission. Moreover, also assert the existence of the EKC hypothesis between patent application and CO_2_ emission in the case of OCED countries
Chen and Lei (2018)	1980–2014	30 countries	Panel quantile regression	Technological innovation reduces CO_2_ emissions substantially in countries with higher emissions levels
Cho and Sohn (2018)	2004–2012	Italy, UK, France, and Germany	LMDI	Environmental-related patents help to reduce carbon emissions from the use of fossil fuels
Salman et al. (2019)	1990–2017	7 ASIAN countries	Quantile regression	Technology innovation significantly and negative impact on CO_2_ emission
Liang et al. (2019)	2000–2015	China’s provinces	SEM and SLM fixed effect	Innovative technologies and the number of patent authorization have significantly reduced the carbon intensity
Luan et al. (2019)	2000–2010	China’s 32 industrial sectors	Linear panel regression	Domestic R&D and technology acquisition reduce the industrial carbon intensity
Cheng et al. (2019)	2000–2013	BRICS	Panel OLS and panel quantile regression	Environmental patents increase CO_2_ emissions due to barriers of patent diffusion. GDP per capita has a significantly positive impact on CO_2_ emission
Khattak et al. (2020)	1980–2016	BRICS	CCEMG technique	Innovation impedes Co_2_ emission through energy consumption
Shahbaz et al. (2020)	1984–2018	China	BARDL	Technological innovations are found to help reduce CO_2_ emissions
Wang and Zhu (2020)	2001–2017	China	Spatial econometric model	Renewable technology innovation facilitates CO_2_ emission abatement, while fossil energy technology innovation is ineffective in reducing CO_2_ emission
Töbelmann and Wendler (2020)	1992–2014	EU-27 countries	GMM	Environmental innovation reduces CO_2_ emissions, while general innovation does not reduce emissions
Huang et al. (2020)	2015–2020	China and Australia 468 sectors	SDA	Developed country helps developing country in Co_2_ emission reduction through the trade of technological innovation
Khan et al. (2020)	1990–2017	G-7 countries	AMG and CCEMG	Examined the nexus among trade, income, innovation, renewable energy, and carbon emission
Chen and Lee (2020)	1996–2018	96 countries	Spatial econometric model	Technological innovation has no significant effect on Co_2_ emission globally
Chaudhry et al. (2021)	1995–2018	East-Asia and Pacific	Dynamic common correlated effect	Technological innovation reduces environmental pollution in the long run
Erdogan (2021)	1992–2018	BRICS	Dynamic common correlated effect	Find out innovation improves environmental quality in the long run for BRICS countries
Ding et al. (2021)	1990–2018	G-7 countries	CS-ARDL	Linked cointegration among international trade, environmental innovation, GDP, renewable energy, and Co_2_ emission

Notes: *OECD*, Organization of Economic Cooperation and Development; *BRICS*, Brazil, Russia, India, China, South Africa; *EU*, European Countries; *GMM*, generalized method of moment; *SEM*, structural equation modeling; *STIRPAT*, stochastic impacts by regression on population, affluence, and technology; *LMDI*, logarithmic mean Divisia decomposition index; *SLM*, spatial lag model; *SEM*, spatial error model; *OLS*, ordinary least square; *CCEMG*, common correlated effects estimation of heterogeneous; *BARDL*, Bayesian auto-regressive distributed lags; *CS*-*ARDL*, cross-sectionally augmented autoregressive distributed lag; *CCEMG*, common correlated effects mean group; *AMG*, augmented mean group

In conclusion, we found two insights from Table 1. First, different environmental technologies are adopted to reduce CO_2_ emission, including the patent for carbon-free energy technologies, research and development in the energy sector, and renewable technology innovations. Second, most studies found that green technology patents or innovation in energy production reduce CO_2_ emissions in the sample of countries/provinces — China, OECD, developed countries, G-7 countries, European Union, and East-Asia and Pacific.

### Renewable energy consumption and CO_2_ emissions

This section presents the nexus between energy consumption and CO_2_ emissions using the past literature. Ito (2017) examines the long-run relationship between renewable and non-renewable energy, economic growth, and CO_2_ emissions for 42 developing countries over 2002–2011 and found that renewable energy reduces carbon emissions in the long run. Zoundi (2017) points out the negative correlation between renewable energy and CO_2_ emissions in a study covering 25 African countries from 1980 to 2012. In contrast, Attiaoui et al. (2017) studied the causal relationship between renewable energy, CO_2_ emissions, and economic growth for African countries for the period 1990 to 2011 and found bi-directional causality between renewable energy, GDP, and CO_2_ emission. A study in Pakistan for a period from 1990 to 2014 found that renewable energy consumption reduces CO_2_ emissions in the long run (Waheed et al. 2018). Chen et al. (2019) found a mixed impact of renewable energy on CO_2_ emissions across different Chinese regions, while Caglar (2020) found that renewable energy reduces carbon emissions in the nine green countries using the bootstrap ARDL model. Abid et al. (2021) also found that renewable energy and technology helped reduce CO_2_ emissions in Pakistan for the period 1990 to 2017. From the literature discussion, it is evident that renewable energy consumption reduces CO_2_ emissions.

### Economic growth and CO_2_ emissions

This section presents the nexus between economic growth and CO_2_ emission using the past literature. The traditional environmental Kuznets curve (EKC) hypothesis in the GDP-carbon emission model argued the existence of an inversed U-shaped relationship between environmental pollution and economic growth. Selden and Song (1994) tested an inverted U-shaped relationship between CO_2_ emission and GDP growth for low middle- and high-income countries for the period 1973–1984. The validity of the EKC hypothesis for ASIAN-5 countries using panel quantile technique for the period 1981–2011 was tested by Zhu et al. (2016). Narayan and Narayan (2010) verified the EKC hypothesis for sample 43 developing countries for the period 1980–2004. Begum et al. (2015), using the data from Indonesia from 1971 to 2007, did not find a causal relationship between carbon emissions and GDP growth, while Ahmed et al. (2019) found that economic growth reduces carbon emissions long run for Croatia using the ARDL model. Solarin et al. (2017) supported an inverted U-shaped relationship between GDP growth and CO_2_ emission for India and China during the period 1965–2013. Mahalik et al. (2018), observed a long-run relationship among CO_2_ emission, energy consumption, GDP per capita, and income inequality for BRICS countries during 1980–2013. Padhan et al. (2019) tested the influence of GDP per capita on CO_2_ emission in the presence of energy consumption and found that GDP per capita energy consumption induces CO_2_ emission in the long run for the next 11 countries during 1971–2013. Using the data from South Asian countries for the period 2000–2018, Ahmed et al. (2022) found that clean energy consumption and green technology upsurged green economic growth, which leads to better environmental health. This review concludes there exists inversed U-shaped relationship between GDP and carbon emission.

### Globalization and CO_2_ emissions

This section reviews the literature on the nexus between globalization and CO_2_ emission. Several empirical studies have explored the connection between globalization and environmental pollution (Allena and Fracchia 2017; Shahbaz et al. 2018, 2020; Wang et al. 2019). Jorgenson et al. (2006) investigated the negative effect of global economic activities on environmental consequences. Shahbaz et al. (2019) examined the relationship between globalization and carbon emissions for the period 1970 to 2012 and found that globalization stimulates CO_2_ emissions in India. Acheampong et al. (2019) studied the association between foreign direct investment (FDI), globalization and carbon emissions in 46 sub-Saharan African countries during the period 1980 to 2015 and identified that globalization weakens environmental quality. Ahmed et al. (2019) found that globalization has no significant effect on the ecological carbon footprint in Malaysia. Sabir and Gorus (2019) analyzed the causal effect of globalization on environmental degradation in a sample of South Asian countries and found that globalization increased environmental degradation for the period 1975–2017. Zaidi et al. (2019a) delved into the association among globalization, financial development, and CO_2_ emission for the OECD countries from 1990 to 2014. Khan and Ullah (2019) investigated the cointegration between globalization and CO_2_ emissions in Pakistan using the autoregressive distributed lag (ARDL) model and found globalization upsurges carbon pollution. For Saudi Arabia, Xu et al. (2018) found bidirectional causality between globalization and CO_2_ emissions for the period from 1971 to 2016. Zaidi et al. (2019b) probed the cointegration among financial development, globalization, and CO_2_ emissions in Asia Pacific Economic Cooperation (APEC) nations from 1990 to 2016 and found that globalization Granger causes carbon emissions. Balsalobre-Lorente et al. (2020) tested the long-run association among economic growth, international tourism, globalization, energy consumption, and carbon emissions for OECD countries in 1994–2014 and found that globalization reduces carbon emissions out of tourism. Literature shows that globalization is detrimental to the environment; however, local environmental regulation could play a critical role in mitigating the detrimental effect of globalization on the environment.

### Urbanization and CO_2_ emissions

This section examines the nexus between urbanization and CO_2_ emission using the past literature. Wang et al. (2016) investigated the causal association among urbanization, energy use, and carbon emission for the Asian countries and found that a 1% increase in urban population results in a 0.20% rise in CO_2_ emission. Behera and Dash (2017) investigated the cointegration between urbanization, energy consumption, and carbon emission in the South and Southeast Asian region during 1980–2012, and the result revealed that urbanization leads to higher environmental degradation in the long run. Salahuddin et al. (2019), using data from 44 sub-Saharan African countries from 1984 to 2016, found that urbanization was positively associated with CO_2_ emissions in the long run whereas negatively in the short run. Hashmi et al. (2021) examined the relationship between urbanization paths and CO_2_ emissions in East Asian countries for the period 1971–2014 and found that urbanization improves environmental health in the long run, while energy intensity and economic growth reduce environmental performances. Using quantile regression, Lee et al. (2022) explored the relationship between urbanization and greenhouse gas emissions for the selected 48 Belt and Road Initiative (BRI) countries from 1984 to 2017 and found that urbanization reduced environmental health.

On the whole, literature portrays a mixed impact of economic growth and globalization on CO_2_ emission, which is moderated by higher renewable energy consumption, rapid urbanization, and foreign direct investment. While environmental technology and innovation could be a mitigation strategy to reduce environmental pollution, not many studies have focused on this.

## Methodology

### Data

Table 2 provides a summary of the variable used in the study. This paper uses data from 14 developing countries^3^ from the Asia region for a period from 1990 to 2018 to examine the effects of environmental technology on the reduction of CO_2_ emission after controlling for renewable energy consumption, economic globalization, economic growth, and urbanization. Variables have been selected based on past literature that supported our assumption that decarbonization is possible through green technology innovation, which contributes to environmental sustainability in the economy (Abid et al. 2021, 2020; Ahmed et al. 2022; Lee et al. 2022).

**Table 2:** List of variables, descriptions, and data sources

Variable	Definition	Data sources
CO_2_	CO_2_ emissions (metric tons per capita)	WDI, World Bank (2020)
EC	Energy use (kg of oil equivalent per capita)	WDI, World Bank (2020)
REC	Renewable energy use (kg of oil equivalent per capita)	Authors’ calculation
GDP	Gross Domestic Product (constant 2010 US$)	WDI, World Bank (2020)
GLOB	KOF economic globalization index	KOF Swiss Economic Institute (2020)
URB	Urban population (% of total population)	WDI, World Bank (2020)
PAT	Environmental related technologies (patents)	OECD 2020

Note: *WDI*, World Development Indicators; *OECD*, Organization of Economic Cooperation and Development

We used macro-indicators for analysis such as CO_2_ emission per capita as a proxy for environmental degradation (CO_2_); total energy consumption per capita (EC); total renewable energy consumption per capita (REC); KOF index of economic globalization (GLOB); percentage of urban population to the total population (URB); and the number of environmental-related technologies patent applications (PAT). We used an environmental-related technology patent as an environmental innovation, suggested by Smith (2005). Data was collected from various sources such as World Development Indicators (WDI) of World Bank (2020), KOF Swiss Economic Institute (2020), Organization for Economic Co-operation and Development (OECD 2020).

**Fig. 2:**
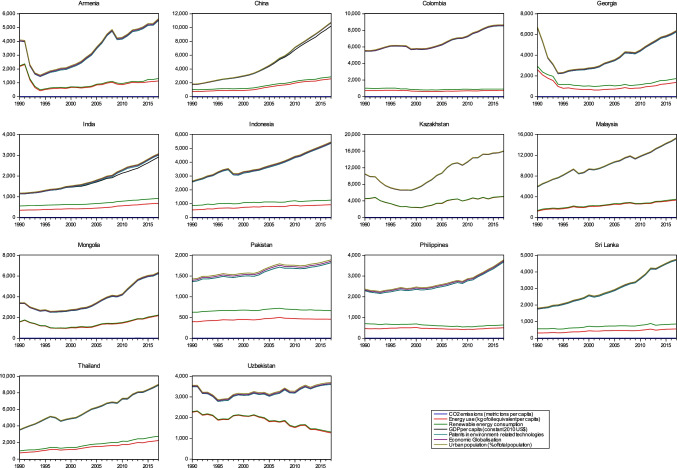
Time-series trends of selected variables across sample countries from 1990 to 2018

The study period and sample selection were based on the data availability of the selected variables. Figure 2 presents the time-series trends of selected variables of individual countries’ samples. It shows the increasing trends of CO_2_ emission over the period via-a-vis other variables in most sample countries. Furthermore, econometric analysis will help us to understand the likely impact of environmental innovation and renewable energy consumption on reducing CO_2_ emission over the period.

### Methods

This study investigates the impact of environmental innovation, globalization, and renewable energy consumption on CO_2_ emission, considering the vital role of economic growth and urbanization in the carbon emissions framework for developing Asian economies. Our model formulation is based on previous recent studies, including Míguez et al. (2018); Zhang et al. (2017); Álvarez-Herránz et al. (2017); Rauf et al. (2018); Mert and Bölük (2016); and Shahbaz et al. (2020); Behera and Pozhamkandath Karthiayani (2021).

1 $${CO}_{2}=f\left(EC, GDP, GLOB, TECH, URB\right)$$

In Eq. (1), CO_2_ denotes carbon emission per capita, EC refers to energy consumption per capita, GDP refers to gross domestic product per capita, GLOB refers to economic globalization index, PAT refers to environmental technologies, and URB refers to the percentage of the urban population of the total population.

2 $$CO_{2it}=\phi_0+\delta_1EC_{it}+\delta_2GDP_{it}+\delta_3GLOB_{it}+\delta_4PAT_{it}+\delta_5URB_{it}+\xi_{it}$$

The current study also examines whether the relationship between economic growth and carbon emissions is inverted U-shaped; therefore, we include a squared term of real GDP per capita into the carbon emissions function. Furthermore, we use an interaction term $GDP_{it} *PAT_{it}$ to show the partial effect of technological innovation on environmental health through the production channel.

3 $$CO_{2it}=\phi_0+\delta_1EC_{it}+\delta_2GDP_{it}+\delta_3GDP_{it}^2+\delta_4GLOB_{it}+\delta_5PAT_{it}+\delta_6URB_{it}+\delta_7({GDP_{it}}^\ast PAT_{it})+\xi_{it}$$

The energy (EC) sensitive carbon emissions coefficient is expected to be positive ($\delta_{1} > 0$) if total energy demand improves the environment or reduces environmental degradation. The relationship between economic growth and carbon emissions is inverted U-shaped if $\delta_{2} > 0$, and $\delta_{3} < 0$. The inverted U-shaped association indicates the presence of the environment Kuznets curve hypothesis. The level of globalization could reduce carbon emissions and enhance environmental quality, so the coefficient is expected to be negative ($\delta_{4} < 0$). As environment-related technology helps improve environmental quality, the relationship between technological innovation and carbon emissions is anticipated to be negative ($\delta_{5} < 0$). The coefficient of urbanization is expected to be positive ($\delta_{6} < 0$) because urbanization could increase environmental pollution. The interaction term $GDP_{it} *PAT_{it}$ shows the joint effect of both GDP and technology on carbon emission if its coefficient is negative ($\delta_{7} < 0$), then technologies reduce CO_2_ emission through the production process.

## Results and discussion

### Summary statistics and correlation

Table 3 presents descriptive statistics and pair-wise correlation of variables from 14 developing Asian countries from 1990 to 2018. The result shows that the mean per capita CO_2_ emission is 3.328 metric tons, while the minimum and maximum values vary between 0.223 and 18.296. The mean per capita energy consumption (EC) is 1257.3 kg, while minimum and maximum values range between 318.4 and 5249.4 kg, respectively. The mean per capita renewable energy consumption (REC) is 192.7 kg, while minimum and maximum values vary between 24.078 and 533.2 kg, respectively. The mean patents related to environmental technologies (PAT) are 13.8. The mean economic globalization index (GLB) is 50.1, while minimum and maximum values vary between 14.7 and 81.1, respectively. The mean urban population to total population (URB) is 48.3%, while minimum and maximum values range between 18.2% and 80.7%, respectively. Results show a high standard deviation in all the variables, indicating high variability among countries in terms of energy consumption and adoption of technology associated with CO_2_ emission. The pair-wise correlation results show CO_2_ emission is positively correlated with EG, GDP, URB, GLB, and PAT while it has a negative relationship with REG. But the simple association between two variables cannot provide an exact relationship between them, and it requires an econometric analysis, which is discussed in the following sub-sections.

**Table 3:** Descriptive statistics and pair-wise correlation (1990–2018)

	CO_2_	EC	REC	GDP	GLOB	PAT	URB
Mean	3.328	1257.353	192.758	3307.806	50.122	13.854	48.330
Maximum	18.296	5249.416	533.247	12,096.81	81.181	479.646	80.792
Minimum	0.223	318.380	24.078	575.501	14.743	0.000	18.196
Std. Dev	3.444	1001.39	109.396	2484.616	14.308	57.261	15.940
Obs	406	406	406	406	406	406	406
CO_2_	1						
EC	0.942***	1					
REC	− 0.421***	− 0.361***	1				
GDP	0.643***	0.696***	− 0.055	1			
GLOB	0.121**	0.159***	0.119**	0.448***	1		
PAT	0.144***	0.128**	0.150***	0.140***	− 0.078	1	
URB	0.371***	0.389***	− 0.387***	0.557***	0.307***	− 0.008	1

Note: ***p < 0.01, **p < 0.05, *p < 0.1

### Results of cross-sectional dependency

We estimated two types of panel cross-section dependence (CD) test — Breusch-Pagan LM, and Pesaran’s CD to examine the correlation between samples and reported the result in Table 4. Because panel data usually encounters the problem of cross-sectional dependency due to mutual shocks and unobserved factors among sample units that need to be removed before regression estimation (Pesaran 2007; Behera and Dash 2018). Similarly, many studies have found a cross-sectional dependence while estimating environmental quality using country-level data (Ahmed et al. 2022; Abid et al. 2020; Chen and Lee 2020). Our results show that the null hypothesis of “cross-sectional dependency” is strongly rejected at the 1% significance level, indicating a cross-sectional dependency among that sample. The result implies that a shock in one developing Asian economy tends to move to other economies.

**Table 4:** Cross-sectional dependence test

Variable	Breusch-Pagan LM	Pesaran CD
CO_2_	19.911*	19.48*
EC	57.613*	9.66*
REC	117.42*	3.07*
NREC	69.37*	14.48*
GDP	226.95*	46.24*
GDP^2^	215.56*	46.30*
GLOB	16.814*	33.39*
URB	93.692*	15.15*
PAT	9.177*	7.87*

Note: ***p < 0.01, **p < 0.05, *p < 0.1

### Results of panel unit-root test in the presence of cross-sectional dependency

Table 5 shows the panel unit-root test results. In this study, we performed both first-generation and second-generation unit-root tests to check the stationarity of the series, which is necessary for cointegration analysis. The first-generation unit-root test comes from Breitung and Das (2005), Levin et al. (2002), and Im et al. (2003), while the second-generation unit-root test comes from Pesaran (2007) CIPS. Several existing studies explained that the first-generation unit-root test could not provide a robust estimate in the presence of cross-sectional dependency because of the influential properties that mislead the null hypothesis of cross-sectional independence (Behera and Dash 2018; and Ahmed et al. 2022). Therefore, Pesaran (2007) proposed a test called cross-sectional Im, Pesaran, and Shin (CIPS) as this test will perform stationarity by taking into account the problem of cross-sectional dependency.

**Table 5:** Panel unit root test

	CO_2_	EC	REC	GDP	GDP^2^	GLOB	URB	PAT
Levin	0.367	− 0.750	0.344	0.387	1.546	4.365	0.043	0.327
IPS	0.654	0.371	0.1229	5.728	6.705	− 0.125	0.893	0.656
Breitung	− 0.807	1.660	− 0.295	1.232	1.258	3.238	− 9.823*	2.105
CIPS	− 2.038	− 1.868	− 2.019	− 2.079	− 2.074	− 2.474	− 0.439	− 0.874
1st difference								
Levin	− 13.375*	− 8.846*	− 15.263*	− 6.813*	− 7.310*	− 11.167*	− 3.642*	− 23.749*
IPS	− 14.047*	− 10.71*	− 16.701*	− 6.829*	− 7.079*	− 10.442*	− 2.179**	− 9.902*
Breitung	− 10.018*	− 7.297*	− 9.901*	− 2.654*	− 2.592*	− 4.564*	− 1.314***	− 15.481*
CIPS	− 4.636*	− 4.684*	− 5.437*	− 3.491*	− 3.452*	− 5.133*	− 4.325**	− 6.117*

Note: *p < 0.01, **p < 0.05, ***p < 0.1

Table 5 reports the CIPS test results, with a null hypothesis of “non-stationary series”. Results reveal that all the series are non-stationary at a level, and series become stationary at the first difference by rejecting the null hypothesis at the 1% significance level, which indicates that all the series are integrated of order 1, i.e., I (1).

### Results of panel cointegration tests

Table 6 presents the long-run relationships among variables using cointegration techniques under the condition that series are stationary at the first difference. In this study, we applied both first-generation and second-generation cointegration models. The first-generation cointegration models include Pedroni (2004); Kao (1999); and Maddala and Wu (1999), while the second-generation include Westerlund and Edgerton (2007). We applied the second-generation cointegration model because when the data suffers cross-sectional dependence, it provides a robust estimate by controlling cross-sectional dependency among variables (Behera and Dash 2018).

**Table 6:** Panel cointegration test results

	EC, GDP, GLOB, URB, PAT	REC, GDP, GLO, URB, PAT	REC, GDP,GDP^2^, GLO, URB, PAT	REC, GDP, GLO, URB, PAT, PAT*GDP
Pedroni (2004)				
Panel v weighted statistic	1.8479**	3.3382***	2.6188**	0.5121
Panel σ weighted statistic	− 0.1607	− 0.4273	0.6371	1.2448
Panel ρρ weighted statistic	− 3.7773*	− 4.2125*	− 3.3045*	− 2.3818*
Panel adf weighted statistic	− 6.8459*	− 6.6149*	− 4.3480*	− 2.1269**
Group σ statistic	0.5701	1.2228	1.7457	2.2644
Group ρρ statistic	− 5.104*	− 3.5634*	− 3.6239*	− 2.6492*
Group adf statistic	− 5.416*	− 5.1585*	− 5.8615*	− 2.6692*
KAO (1999)				
Maddala and Wu (1999)				
Modified Dickey-Fuller *t*	− 2.9091*	− 4.2422*	− 2.0319**	− 0.5309
Dickey-Fuller *t*	− 3.8549*	− 5.2914*	− 1.5509**	− 2.4041*
Augmented Dickey-Fuller *t*	0.1306	− 1.0298	− 2.0724**	1.1239
Unadj.Mod.Dickey-Fuller *t*	− 8.9674*	− 10.0365*	− 2.5729*	− 10.1734*
Unadjusted Dickey-Fuller *t*	− 6.2156*	− 7.2422*	− 1.8065**	− 7.3114*
Westerlund and Edgerton (2007)				
G_t_	− 3.171**	− 3.065**	− 3.442*	− 2.685
G_a_	− 0.419	− 7.537	− 7.472	− 5.688
P_t_	− 12.215*	− 14.367*	− 13.984*	− 11.747*
P_a_	− 7.479	− 10.135	− 8.122	− 8.021

Note: *p < 0.01, **p < 0.05, ***p < 0.1

Overall cointegration tests show a long-run co-movement among variables and reject the null hypothesis of no cointegration at below 5% level of significance. So, the results imply that energy consumption, economic growth, urbanization, and environmental technology patent are correlated with CO_2_ emissions in the long run.

### Results of panel long-run regression estimate

Our study estimates the effects of green technology innovation (i.e., number of patents on clean technology) on CO_2_ emission in the long run by controlling per capita GDP, economic globalization, urbanization, consumption of non-renewable energy, and oil consumption. We use three estimation models such as fully modified ordinary least square (FMOLS), fully modified generalized least square (FGLS), and panel-corrected standard error (PCSE), to examine the long-run elasticity of per capita CO_2_ emissions in developing Asia from the period 1990 to 2018.

Table 7 presents the estimation results using four regression specifications. Models 1–3 estimate the impact of per capita energy consumption (EC), per capita GDP (GDP), economic globalization (GLOB), rate of urban population (URB), and the number of the patient on green/clean technology (PAT) on per capita CO_2_ emission (CO_2_). Models 3–6 regressed CO_2_ emission with renewable energy consumption (REC), GDP, GLB, URB, and PAT. In models 7–9, we used GDP square as one of the regressors to estimate the non-linear effects of CO_2_ emissions. In models 10–12, we used an intersection variable (GDP*PAT) as one of the regressors to examine whether economic growth and green technology innovation have CO_2_ reducing effect.

**Table 7:** Panel long run results

LCO_2_: dependent variable												
Independent variables	EC, GDP, GLB, URB, PAT			REC, GDP, GLB, URB, PAT			EC, GDP, GDP^2^, GLB, URB, PAT			EC, GDP, GLB, URB, PAT, GDP*PAT		
	1	2	3	4	5	6	7	8	9	10	11	12
	FMOLS	PCSE	FGLS	FMOLS	PCSE	FGLS	FMOLS	PCSE	FGLS	FMOLS	PCSE	FGLS
LEC	0.8819*	1.2829*	1.1167*									
LREC				− 0.1512*	− 0.2971*	− 0.5936*	− 0.5877*	− 0.5829*	− 0.5829*	− 0.5758*	− 0.5859*	− 0.5859
LGDP	0.0784*	− 0.1031*	0.0922	0.6468*	0.5905*	0.7898*	0.3040*	0.3014*	0.3014**	0.2565*	0.2699*	0.2699**
LGDP^2^							0.0299*	0.0307*	0.0307*	0.0331*	0.0338*	0.0338
LGLOB	− 1.2774*	0.3204*	− 0.068***	− 0.1920*	− 0.2803*	− 0.3314*	− 0.2664*	− 0.2926*	− 0.2926*	− 0.3050*	− 0.2991*	− 0.2991
PAT	0.005*	0.0007**	− 0.00001	− 0.00025	0.0007***	0.003*	0.003*	0.0030*	0.0030*	0.0296*	0.0268*	0.1594***
LURB	− 0.267*	0.1488*	0.1239***	0.4411*	0.5900*	0.1155	0.1261*	0.1369***	0.1369	0.1594*	0.1594**	0.0268*
LGDP*PAT										− 0.0026*	− 0.0027*	− 0.0027**
***R***^**2**^	0.5454	0.9172		0.958	0.3645		0.651	0.7929		0.5927	0.796	

Note: *p < 0.01, **p < 0.05, ***p < 0.1

Models 1–3 results show the effects of EC, GDP, and PAT on CO_2_ emission are positive and statistically significant at a 1% level. The elasticity of CO_2_ emission per capita to per capita EG is less than one in FMOLS while greater than one in PCSE and FGLS, but the coefficient value is very high (varies 0.881–1.282%). We found an unexpected positive association between CO_2_ emissions and PAT, but the coefficient value is small (varies 0.005–00,007%). It is evident that green technology innovation is in the infant stage in Asian countries, and energy consumption (oil use) still is the dominant source of air pollution. Additionally, we found the effects of economic globalization, and urbanization on CO_2_ emission is negative and statistically significant at a 1% level using FMLOS (model 1). It indicates that rapid economic transformation reduces pollution levels, but the result is inconsistent in PCSE (model 2) and FGLS (model 3).

Models 4–6 results show that the effects of REC and GLOB on CO_2_ emission are negative and statistically significant at a 1% level. The elasticity of CO_2_ emission per capita to renewable energy consumption is less than one, and the coefficient value varies from − 0.151 to − 0.593. Similarly, economic globalization has succeeded in reducing CO_2_ emission, and the result is consistent across models and regression specifications. Green technology innovation (PAT) is also ineffective in reducing CO_2_ emissions. Factors such as urbanization and economic growth increase the CO_2_ emission across all models.

The result shows that rapid economic growth and urbanization contribute to an increase in the GHG emissions level. As urbanization and economic growth positively affect economic development and well-being, no country would reduce urbanization and growth for decarbonization. Therefore, urbanization and growth supported by green energy and technological innovation could reduce GHG emissions and contribute to sustainable development. However, the use of coal, fossil fuel, and biomass by different sectors of the economy across the globe is extremely high, and it is unlikely that switching to renewable energy can happen in the near future.

In models 7–9, we regressed CO_2_ emission with similar regressors adopted in models 4–6, except that we added GDP square as one of the regressors. The coefficient of the GDP square is positively associated with CO_2_ emission, indicating that higher income does not lead to the use of emission-free energy. The current study does not support EKC because most of the countries used in the study are in their early stage of development, and the positive effect of GDP on the environment is yet to start in full potential.

Models 10–12 presents the effects of an interaction model (GDP*PAT) on CO_2_ emission by controlling EC, GDP, GLB, and URB and found that the interaction term is negatively correlated with the CO_2_ emission, while the coefficient of environmental technology and GDP is positive and significant. Developing Asian countries can reduce CO_2_ emissions by maintaining minimum technological progress in production. In other words, countries should maintain a certain level of efficiency in production quality or production-based innovation activities to reduce CO_2_ emissions. This result corroborates the finding of Zhang et al. (2017), which suggests that firms must follow environmental innovation at the root level through a network system to reduce CO_2_ emission.

Surprisingly, the result shows a positive relationship between innovation and CO_2_ emission, and the coefficient of CO_2_ emission per patent is between 0.001 and 0.03. This suggests that environmental innovation increases CO_2_ emission in developing Asian economies, which is in line with other studies such as Brandão Santana et al. (2015) and Su and Moaniba (2017). It is challenging to scale our new environmental technology to harness its potential to reduce environmental pollution due to intellectual property rights issues. For instance, Raiser et al. (2017) explained that inventors protect the knowledge of environmental technologies from third parties, which restricts global access to new technology. Furthermore, Dauda et al. (2019) pointed out that green energy technologies might be less effective in reducing carbon emissions in an earlier stage of development due to the overhead cost of technological innovation.

The impact of energy use is significant and positive on CO_2_ emission, revealing that energy use contributes to environmental degradation. The elasticity coefficient of energy use to carbon emission is ~ 0.8 to ~ 1.3, which indicates that a 1% increase in energy use raises carbon emissions by more than 1% on average in developing Asia, suggesting that an increase in energy consumption increases environmental degradation. Furthermore, renewable energy consumption has a negative and significant effect on environmental degradation. The renewable energy-sensitive coefficient is estimated at ~ 0.5, which means that a 1% increase in renewable energy per capita diminishes carbon emission by 0.5% per capita in the developing Asian sample.

GDP is positively associated with carbon emissions without the EKC model. The positive relationship between GDP and CO_2_ emission shows that an increase in GDP leads to a rise in carbon emission. The result indicates that a 1% increase in GDP per capita leads to CO_2_ emission by 0.8% on average. However, the current study does not support the EKC hypothesis for GDP and CO_2_ emission. It may be because the countries in the study are yet to reach their peak, after which the CO_2_ emission will decline with an increase in GDP.

In our sample study, the long-run relationship between globalization and carbon emission is negative. The findings affirm that a 1% increase in economic globalization leads to an average ~ 0.2 to ~ 2.2% fall in carbon pollution for developing Asian countries. The empirical evidence shows that urbanization has an increasing effect on CO_2_ emissions, and the estimated coefficient for urbanization varies from 0.02 to 0.6. In other words, a 1% increase in the urban population leads to a 0.6% increase in carbon emission.

Overall results conclude that economic growth, urbanization, and oil consumption are the main source of CO_2_ emissions. Our result is consistent with past studies Poumanyvong and Kaneko (2010), Bekhet and Othman (2017), Lu, W. C. (2017), Pata (2018), Khattak et al. (2020), Abid et al. (2021), and Mohsin et al. (2021). Economic globalization, renewable energy consumption, and green technology adoption associated with higher economic development are the pertinent factors to reduce CO_2_ emissions. Although very few studies have adopted green technology innovation (patent) to examine the likely effects of pollution, few past studies argued that environmental innovation has no significant impact on carbon pollution in the long run (Chen and Lee 2020). The spillover effects of technological innovation can bring healthier environmental quality in developing countries (Ling et al., 2015). Chen and Lee (2020) argue that the indirect or spillover effects of technological innovation are heterogeneous among different income groups of countries. Green technology does not reduce CO_2_ emission in low-technology countries, while green technology has the potential to reduce CO_2_ emission in countries with well-developed environmental technologies, as shown in model 4.

### Results of panel Granger causality

**Fig. 3:**
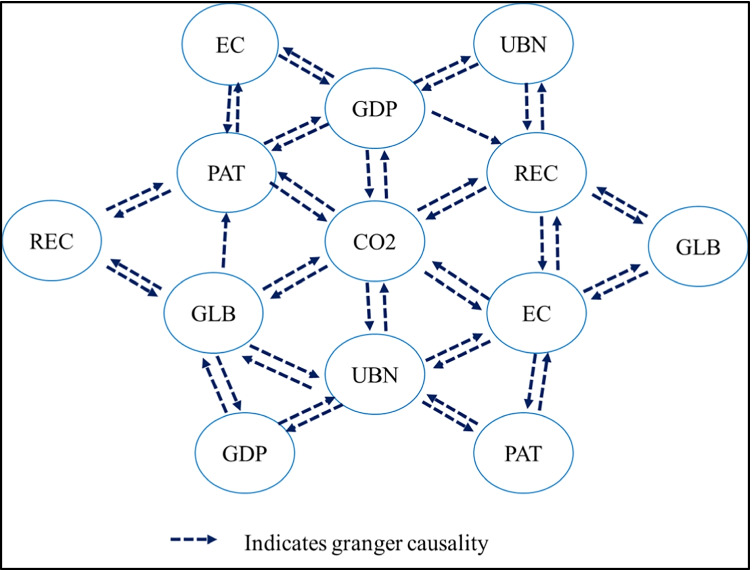
Graphical Dumitrescu-Hurlin Granger causality tests. Source: Author(s) estimation

The study also examines the possible causal relationships between variables and explores its direction of causality, in the long run, using Dumitrescu and Hurlin (2012) panel Granger causality test. Figure 3 presents the direction of causality between variables.^4^ Understanding the causality between variables will predict the possible confounder of environmental degradation and appropriate policy that could be taken to mitigate those environmental pollutants. Our result shows the presence of bidirectional causality running from environmental technology, globalization, GDP, renewable energy, energy, and urbanization to CO_2_ emission and vice-a-versa in developing Asia. Furthermore, a bidirectional causality runs from environmental technology, total energy, globalization, and urbanization to economic growth and vice-a-versa.

Moreover, unidirectional causality runs from economic growth to renewable energy, while no reverse causality is noticed from renewable energy consumption to economic growth. Similarly, bidirectional causality between renewable energy consumption and green technology innovation is good for reducing emissions through environmental technology. Globalization also plays a vital role in environmental innovation. These results are supported by Brandão Santana et al. (2015); Lu (2017a, b), for G7 countries Ding et al. (2021) and Khan et al. (2020), and for APEC Shahbaz et al. (2018), which confirmed the existence of bidirectional causality running from GDP growth, renewable energy, urbanization, technology, and trade to CO_2_ emissions. Overall, results indicate that globalization, renewable energy consumption, and green technology innovation could be a channel for improving environmental quality and reducing air pollution in developing countries of Asia.

## Conclusions and policy implications

This paper uses relevant data from 14 developing countries in Asia from 1990 to 2018 to examine the potential impact of environmental innovation on CO_2_ emission by controlling for globalization, urbanization, economic growth. We use the number of environmental-related technologies patents to measure environmental innovation. For empirical estimation, we have employed a panel long-run regression model — FMOLS, PCSE, and FGLS to estimate the elasticity of CO_2_ emission. We use Dumitrescu and Hurlin (2012) Granger causality tests to test the causal association among variables.

Our result shows the presence of bidirectional causality running from environmental technology, globalization, GDP, renewable energy, total energy, and urbanization to CO_2_ emission and vice-a-versa in developing Asia. Similarly, bidirectional causality between renewable energy consumption and green technology innovation reduces emissions through environmental technology. Our results show that renewable energy consumption and economic globalization have a more significant impact on the reduction of CO_2_ emissions, while environmental technology innovations play a meager role in reducing emissions only when economic growth support those type of investment. Furthermore, we found urbanization, oil consumption, and economic growth is detrimental to the environment, which is also evident in past studies. Green energy and technological innovation and scaling it to developing countries could play a significant role in decarbonization, mitigating climate change, and contributing to sustainable development. Therefore, the countries should invest in renewable energy and environmental innovation aligned with the growth.

Although our study has many novelties in terms of the adoption of new variables — environmental patents, which indicates the new path of environmental sustainability through decarbonization, it has few limitations. First, the most recent data on environmental quality (i.e., CO_2_ emission (GHG)) was not available. Using the latest year data (i.e., 2020 and 2021) could show whether COVID-19 lockdown had any impact on reducing emissions. Second, the current study did not use green technology/innovation investment that might directly impact CO_2_ emission. Third, government fiscal spending on CO_2_ abatement and governance structure can provide a better insight on the issue of the decarbonization policy of the economy. These limitations are due to a lack of data, especially in developing countries, that could be addressed in future research.

## Data Availability

The data used for this study is available in the public domain for research purposes.

## Footnotes

1 Top five GHG emitting countries includes China, USA, India, Russian Federation, and Japan.

2 Countries used in this study include China, India, Indonesia, Thailand, Kazakhstan, Malaysia, Pakistan, Philippines, Uzbekistan, Colombia, Sri Lanka, Mongolia, Georgia, Armenia.

3 Our enalysis includes 14 developing Asian countries due to availability of annual data point. The selected countries are Armenia, China, Colombia, Georgia, India, Indonesia, Kazakhstan, Malaysia, Mongolia, Pakistan, Philippines, Sri Lanka, Thailand, and Uzbekistan.

4 The Granger causality test has been done through VAR automatic lag order selection criterion. The coefficient and probability result has not reported here, and it will be available upon request.
